# Strategies for Assessment and Decision Making in Complex Limb Fracture Management: A Review

**DOI:** 10.7759/cureus.97913

**Published:** 2025-11-27

**Authors:** Nur Amelia S Shaharudin, Olivia Dunseath, Nur Aina Azmi, Ning Yee Aun, Alexandra Foley

**Affiliations:** 1 Trauma and Orthopaedics, Barts Health National Health Service (NHS) Trust, London, GBR; 2 Critical Care, Rotherham General Hospital, Sheffield, GBR; 3 Psychiatry, Hertfordshire Partnership University Foundation Trust, Hertfordshire, GBR

**Keywords:** advanced trauma life support (atls), british orthopaedic association standard for trauma (boast), complex limb injuries, limb salvage versus amputation, open fracture, review of literature

## Abstract

Complex limb injury (CLI) denotes extremity trauma, involving damage to bone, soft tissue, blood vessels, and nerves, most often caused by high-energy blunt impact. These injuries necessitate urgent, structured assessment and close collaboration between multiple specialties (i.e., orthopaedics, plastics, and vascular). Initial priorities focus on preserving life, controlling bleeding, and restoring circulation, followed by stabilising fractures and preventing complications such as compartment syndrome. Deciding whether to attempt limb salvage or proceed to amputation remains one of the most difficult judgments in trauma care. While scoring systems such as the Mangled Extremity Severity Score (MESS), Nerve injury, Ischemia, Soft tissue, Skeletal injury, Shock, Age of patient (NISSSA), Limb Salvage Index (LSI), Predictive Salvage Index (PSI), and Ganga Hospital Open Injury Severity Score (GHOISS) can help guide decisions, their accuracy is limited in modern practice, and no single tool has gained universal acceptance.

Evidence indicates that both salvage and amputation can achieve similar long-term functional or quality of life outcomes, although salvage is often associated with a heavier treatment burden. Current guidance emphasises individualised decision-making, taking into account the patient’s overall health, preferences, and circumstances, and ensuring that discussions involve both the patient and their family. There is still a clear need for more reliable, validated, and patient-centred decision aids that incorporate advances in trauma care and prioritise long-term quality of life.

## Introduction and background

Complex limb injury (CLI), sometimes referred to as a mangled limb injury, refers to injuries of the extremities with disruption of the anatomy affecting bone, muscle, tendon, nerve, vasculature, and soft tissues, which can compromise limb viability and function [1]. An important contributing factor in CLI is the mechanism of injury, with blunt trauma, such as motor vehicle crashes and industrial accidents, being the leading cause [2-6]. The mechanism by which injury is sustained is important, as the energy absorbed is translated into the degree of destruction of the extremities [2]. A thorough yet focused assessment of patients presenting with CLI is vital, as promptly identifying both life-threatening and limb-threatening injuries directly influences management decisions and impacts patient morbidity.

The management of such injuries requires a multidisciplinary approach across multiple specialities, including (but not limited to) orthopaedics, vascular, plastics, critical care, and rehabilitation medicine. Such a meticulous approach is required, as one of the most critical decisions is whether to proceed with limb salvage or amputation, a choice for which no clear consensus currently exists [7-9]. This comprehensive narrative review aims to explore the various factors that influence the decision-making process in managing CLI.

## Review

Initial assessment of complex limb injuries

According to the Advanced Trauma Life Support (ATLS) principles, trauma patients with CLI should be transferred to a Level 1 trauma centre equipped with the necessary resources to manage such injuries effectively [10]. As with any trauma patient, a thorough assessment begins with a primary survey, followed by any necessary resuscitation efforts. In practice, the principle of “life over limb” is reflected in established trauma priorities: immediate control of life-threatening haemorrhage, restoration of adequate circulation and perfusion, and consideration of timely fasciotomy when there is a recognised risk of compartment syndrome. Pre-existing guidance on the initial management of trauma patients should be adhered to, including the ATLS principles [10], British Standards for Trauma (BOAST) guidelines [11], and National Institute for Health and Care Excellence (NICE) guidelines [12]. In cases of major haemorrhage, bleeding should be controlled with direct pressure where possible. If direct pressure fails to control haemorrhage, evidence supports the use of a tourniquet as a temporising measure pending definitive haemostasis [10,13]. Following physiological stabilisation during the primary survey, proceed to focused assessment of the affected extremities.

The CLI initial assessment includes identifying and appropriately managing arterial injuries, peripheral nerve injuries, and open fractures [8]. Assessment of vascular injury is essential, as the risk of vascular insult increases proportionally with the severity of high-energy trauma [14,15]. An estimate of five percent of open fractures are associated with a vascular injury requiring surgical repair, with an amputation rate of around 16% [15,16]. Severe open tibial fractures specifically carry a 9% incidence of vascular injury [17]. Knee dislocations, particularly posterior dislocations, pose a significant risk to the popliteal artery, with studies indicating that up to one in five of these patients may ultimately require amputation [18]. In paediatric cases, vascular injury is a particular concern in supracondylar fractures, with injury to the brachial artery occurring in 8-10% of children with a Gartland III supracondylar fracture [19,20].

During the primary survey, assessing pulses and capillary refill time supported by adjuncts such as a handheld Doppler or an ankle-brachial pressure index can help identify vascular injury [21]. The presence of hard or soft signs guides the management of vascular injuries. Table 1 outlines these signs in the context of extremity vascular injury, providing a valuable tool for the early identification and management of acute vascular injury in trauma patients [15]. The presence of any hard sign has a sensitivity of 92-95% for detecting vascular injury requiring surgical intervention and typically warrants immediate operative management without delay for further imaging, such as a computed tomography angiogram (CTA) [14]. In contrast, the presence of a single soft sign is associated with an approximate 10% risk of vascular injury, while two or more soft signs raise this risk to around 25% [22].

**Table 1:** The hard and soft signs of vascular injury to the extremities Adapted from Stefanou et al. [15]. Tables are published under a Creative Commons License.

Hard signs	Soft signs (or subtle signs)
Active pulsatile or massive bleeding	History of arterial bleeding
Rapid expanding or pulsating haematoma	Proximity related injury
Bruit/thrill	Neurologic findings from nerve adjacent to a named artery
Evidence of arterial insufficiency and ischaemia	Haematoma over a named artery
-	Diminished unilateral distal pulse

In cases of a devascularized limb, initial management typically involves the placement of a temporary intravascular shunt and skeletal stabilisation (i.e., application of external fixation). Once perfusion is restored, definitive vascular repair, soft tissue assessment, and reconstruction can be performed [8,14,15]. If no hard signs are present during the primary survey, soft signs should be evaluated during the secondary survey. In cases of suspected injury, a CT angiogram is the gold standard for confirming the diagnosis and guiding further management. It is essential to recognise that physical examination alone may be insufficient to exclude a limb-threatening injury in the absence of apparent clinical signs [14,15].

In recent years, there has been an ongoing debate over whether the presence of hard signs alone should remain the primary indication for emergency surgical exploration in CLI. Romagnoli et al. analysed data from 1,910 patients in the American Association for the Surgery of Trauma PROspective Observational Vascular Injury Treatment (PROOVIT) registry and found that patients with ischaemic signs who underwent surgical exploration required more blood transfusions within the first 24 hours [23]. However, there was no significant difference in amputation rate, reintervention rate, length of hospital stay, or mortality compared to those diagnosed via CTA. Their study suggests that relying solely on hard and soft signs for vascular injury may be outdated. Instead, they propose a more clinically useful approach that prioritises haemorrhagic and ischaemic signs to guide investigation and management [23,24]. 

Many of these injuries are associated with fractures, where an accompanying wound classifies them as open fractures. When appropriate, a reduction manoeuvre should be attempted to improve fracture alignment, which can help reduce pain and bleeding to some extent. The BOAST guidelines provide clear recommendations for managing open fractures, including the removal of visible gross contamination, photographing the wound, and covering it with saline-soaked gauze. It is crucial not to wash out or irrigate the wound in the Emergency Department [25]. When appropriate, fracture realignment should be attempted, followed by splinting to maintain the alignment. The guidelines also recommend administering a broad-spectrum antibiotic within an hour of injury and providing a tetanus booster if the patient has not had a recent dose. Clear communication with the pre-hospital team is essential to ensure the initial or subsequent doses are administered appropriately [25]. The BOAST guideline recommends a time estimate for the first debridement, depending on the mechanism of injury and exposure to contamination [25]. Once the patient can be assessed intraoperatively, the full extent of the soft tissue damage associated with the open fracture can be appropriately evaluated and graded using the Gustilo-Anderson classification [8,16].

It is important to remain vigilant about compartment syndrome in patients with CLI. A study by McQueen et al. found that 69% of acute compartment syndromes were associated with a fracture, most commonly involving the tibial shaft [26]. The diagnosis of compartment syndrome remains clinical, characterised by signs such as pain disproportionate to the injury despite strong analgesia, firm compartments on palpation, and pain on passive stretch [26]. However, in trauma patients who may be intubated and ventilated, it can be challenging to assess their pain clinically; in such cases, needle compartment pressure measurements can be used to aid diagnosis. Compartment syndrome is a surgical emergency that needs urgent intervention, specifically fasciotomy [27]. Compartment syndrome can also emerge as a complication following limb reperfusion [28]. Often, vascular surgeons perform prophylactic fasciotomies in patients who require restoration of circulation in their extremities to prevent this [8,15,28,29]. 

In limb trauma, several factors influence the decision between limb salvage and amputation. However, it is crucial to consider the patient as a whole. While the acute injury and haemodynamic status play a significant role in management decisions, the long-term functional outcome must also be considered. A comprehensive assessment should include the patient’s medical history, their pre-injury functional status, and their social circumstances. Ultimately, if the patient has decision-making capacity, their autonomy must be fully respected. Their family should also be involved throughout the decision-making process to ensure a holistic and patient-centred approach.

Limb salvage vs amputation

Following a thorough assessment and appropriate initial management, the decision between limb salvage and primary amputation is complex. It requires a multidisciplinary approach from various specialities, such as but not limited to orthopaedics, vascular, plastics, critical care, and rehabilitation medicine, as recommended by both BOAST and NICE guidelines [8,9]. At the heart of this process is the patient, who must navigate the impact of this life-changing decision. While various clinical tools can aid decision-making, it is essential for clinicians to prioritise patient involvement, ensuring they are included as much as possible throughout the process. Aravind et al. conducted a qualitative study of 20 patients with type IIIB or IIIC open tibial fractures, exploring their perspectives on amputation versus reconstruction. Despite the small sample size, the study revealed the emotional complexity of the decision and the importance of clear, early communication with not only the patient but also their family members, as many of the patients were too sedated to participate meaningfully in early discussions. It highlights the need for clear, timely communication with both patients and their families to support truly informed decision-making [30]. Both limb salvage and amputation carry their own risks. In the management of chronic limb ischaemia, the most common complications are wound infection in amputees and non-union in limb salvage patients [24]. Patient-specific factors, including pre-existing comorbidities and lifestyle choices such as diabetes and smoking, further exacerbate these risks [24,31].

A systematic review of nine studies by Busse et al. reported comparable long-term functional outcomes and quality of life between patients undergoing primary amputation versus complex limb salvage following severe lower limb trauma. However, limb salvage was associated with a greater treatment burden, involving a greater number of surgical procedures and prolonged hospitalisation. It is essential to note that this review included only a limited number of observational studies, with considerable heterogeneity across the included trials, which may limit the generalisability of its findings [32]. Despite the development of numerous clinical tools to aid in decision-making, there remains no clear consensus or high-quality evidence to establish one tool as superior to others [2,24,33]. As a result, clinical judgment on a case-by-case basis remains a key factor in guiding decisions, as equivalent functional outcomes can be achieved in both limb salvage and amputation [24].

There is a plethora of available scoring systems that aim to aid the decision-making process. The Mangled Extremity Severity Score (MESS) is a scoring system that focuses on four variables: skeletal and soft tissue injury, limb ischaemia, shock, and patient age, to predict the need for amputation following a CLI [34]. Several authors have concluded and recommended that a MESS score greater than 7 is an indication for amputation [9,24]. At the time of its publication in 1990, Johansen et al. achieved 100% accuracy when using MESS and a predictor for amputation [34]. However, in more recent times, with medical advancement in limb salvage since its conception, there has been doubt over the reliability of MESS. Highlighted in a systematic review by Schiro et al., the results of the 1990 study were not reproducible [35]. A modified MESS, which is known as the score for nerve injury, ischaemia, soft-tissue injury, skeletal injury, shock, and age of patient (NISSSA), incorporates a nerve injury component to the widely used MESS. Despite it being more sensitive and specific than its predecessor, there are also criticisms of the scoring system due to its emphasis on loss of plantarflexion in the acute phase, which may be secondary to neuropraxia rather than a more serious indication for a nerve injury [24,35-37].

This highlights the vast number of scoring systems that have been developed over the years to refine and improve upon existing models. Several other scoring systems have been developed to predict the need for primary amputation, including the Ganga Hospital Open Injury Severity Score (GHOISS) [38], Limb Salvage Index (LSI) [39], Mangled Extremity Syndrome Index (MESI) [40], and Predictive Salvage Index (PSI) [41]. A literature review conducted by Akgun Demir et al. [42] summarised the different variables in the scoring systems, as shown in Table 2.

**Table 2:** Summary of the different variables looked at in the different scoring systems used to predict necessity for amputation in CLI ^+(plus)^ Variable included in scoring system, ^- (minus) ^Variable not included in scoring system. CLI: Complex limb injury, MESI: Mangled extremity syndrome index, MESS: Mangled extremity severity score, PSI: Predictive salvage index, LSI: Limb salvage index, NISSSA: Nerve injury, Ischemia, Soft tissue, Skeletal injury, Shock, Age of patient, GHOISS: Ganga hospital open injury severity score. Obtained from Akgun Demir et al. [42].

	MESI	MESS	PSI	LSI	NISSSA	GHOISS
Age	+	+	-	-	+	+
Shock	+	+	-	-	+	+
Warm Ischaemic Time	+	+	+	+	+	+
Bone Injury	+	-	+	+	-	+
Muscle Injury	-	-	+	+	-	+
Skin Injury	+	-	-	+	-	+
Nerve Injury	+	-	-	+	+	-
Deep Vein Injury	-	-	-	+	-	-
Skeletal/Soft Tissue	-	+	-	-	+	-
Contamination	-	-	-	-	+	+
Time To Treatment	+	-	-	+	-	-
Comorbidity	+	-	-	-	-	+

In 2010, the Lower Extremity Assessment Project (LEAP), led by Higgins et al. [43], conducted a multicentre study across Level 1 trauma centres in the United States to validate several established scoring systems, including MESS, PSI, LSI, NISSSA, and the Hannover Fracture Scale-97 (HFS-97) [9,43]. The LEAP study found that these scoring systems were neither accurate nor reliable in predicting the need for amputation, demonstrating low sensitivity [43]. However, it is essential to note that the study did not propose a definitive criterion or guideline for limb salvage versus amputation [9]. This ultimately means that the time-critical decision remains in the hands of clinicians together in a multidisciplinary team, who must consider a wide range of patient-specific factors, many of which are reflected in the various scoring systems. Table 3 provides a summary of each scoring system, variables used, strengths and limitations.

**Table 3:** Summary of each scoring system, variables used, strengths and limitations

Scoring system	Summary	Variables used	Strengths	Limitations
Ganga Hospital Open Injury Severity Score (GHOISS)[38]	The GHOISS was developed and validated by Rajasekaran et al. [38,44]. The score was developed to grade the severity of Gustilo Type III A and B limb injuries without a vascular injury.	- Skin and soft tissue injury (score 1-5) - Skeletal injury (bone and joints) (score 1-5) - Muscle, tendon, and nerve injury (score 1-5)	- The score is explicitly tailored for Type III-B open tibial fractures - Incorporates key orthoplastic variables relevant to reconstruction planning - Has demonstrated comparable specificity and sensitivity in guiding decisions between limb salvage or amputation in both adults and paediatric populations [45,46] - Provides a clear cut-off score (<=14 vs >=17) to assist in decision-making between salvage and amputation - Can be used for fractures without vascular compromise Subsequent studies have demonstrated reliable predictive outcomes when utilising this score [38,44,47,48]	- Explicitly designed for lower limb injuries (tibia), so lacks generalisability to other fracture types - Experience in evaluating soft tissue, bone and neurovascular status is required for accurate scoring. Without this, the score may be biased - Some variables, such as the degree of muscle damage, may only be evaluated intra-operatively, limiting its use in early triage
Limb Salvage Index (LSI) [39]	The LSI was developed from an analysis of 70 limbs with major lower extremity combined orthopaedic and vascular injuries. The authors who created the score reported a 100% correlation between the limb outcome and the threshold score [39].	- Skin injury (score 0-1) - Muscle injury (score 0-2) - Bone injury (score 0-2) - Nerve injury (score 0-2) - Vein injury (score 0-1) - Arterial injury (score 0-2) - Length of warm ischaemia time (score 0-4) Total score range: 0-14 - LSI <6: limb salvage - LSI>= 6: Amputation (primary, secondary or functional)	- Comprehensively considers vascular, skeletal, soft tissue, and neurological damage, plus ischaemia duration - Bosse et al. found LSI to perform significantly better than the MESS and PSI when the scoring systems were applied to all of the limbs in the study, and performed significantly better than did the MESS, PSI< NISSA, and HFS-97 when only type III tibia fractures were considered [36]	- Does not take into account patient demographics, comorbidities, additional injuries, patient baseline or preferences - Developed from a retrospective single-centre retrospective study; therefore, external validation is limited [39] - Only applicable to injuries with combined orthopaedic and vascular injuries - Reduced sensitivity and specificity were described by future authors when analysing the score, - In a prospective study of 88 ischemic limbs, Bosse et al. [36] was unable to replicate the original findings of the LSI; the sensitivity was 83%, and the specificity was 82%.
Mangled Extremity Syndrome Index (MESI) [40]	The MESS score was reported on in 1990 by Johansen et al. [40] by reviewing 25 trauma victims with 26 severe lower-extremity open fractures with vascular compromise.	- Degree of injury to the skeletal and soft tissues (score 1-4) - Presence and duration of shock (score 0-2) - Age of the patient (score 0-2) - Severity and duration of limb ischaemia (score 0-3) If ischaemia time is more than six hours, add 2 points. Johansen et al. reported that a score of 7 or more predicted amputation with 100% accuracy [40]	- Quick and straightforward to use in emergency settings - Encourages a structured assessment of the key injury domains	- More recent studies have shown the score to have a good specificity but poor sensitivity for amputation in modern trauma care [19,36,49,50] - The score is now outdated as it was developed before modern advances in trauma care, such as novel vascular interventions, tourniquets and microsurgical techniques - Does not take into account patient preferences or functional outcomes - Severity of concomitant nerve, venous or soft tissue injuries not adequately accounted for - Not validated for upper limb injuries - Assumes different outcomes in <30s vs 30-50 year olds [51]
Predictive Salvage Index (PSI) [41]	The PSI score was developed by Howe et al. in 1987 [41] following a retrospective review of 676 tibial-fibular fractures and 985 femoral fractures.	- Degree of bone damage (score 1-3) - Level of arterial injury (score 1-3) - Degree of injury to the muscles (score 1-3) - Warm ischaemia time (score 1-3)	- Howe et al. reported a sensitivity of 78% and a specificity of 100% for the PSI score in their patient cohort [41] - Designed to specifically avoid prolonged, unsuccessful attempts at limb salvage in patients with complex lower limb injuries, in particular those with combined vascular disruption	- Subsequent studies have reported a much lower sensitivity and specificity of the PSI score compared to the original findings [52,53] - Similar to the MESS score, it does not take into account advances in trauma care, such as novel vascular interventions, tourniquets and microsurgical techniques - Does not consider patient-specific factors such as comorbidities, patient demographics and patient wishes

Schiro et al., in their previously mentioned systematic review, concluded that scoring systems have limited practicality in predicting functional outcomes or guiding decisions between limb salvage and amputation [35]. NICE guidelines further support this by recommending that an injury severity tool should not be the deciding factor for salvage versus amputation. Still, in a life-threatening haemorrhage situation, an emergency amputation should be considered, and a delayed primary amputation should be performed within 72 hours, given no threat to the patient’s life once decided [8,9].

Future perspectives and recommendations

Given the complexity of decision-making in complex limb injuries and the aforementioned current limitations of existing clinical tools [36], future efforts must prioritise the development of more accurate, evidence-based decision-making aids. Furthermore, a lot of these tools are outdated, failing to incorporate modern advances in trauma care such as novel vascular interventions, tourniquets, and microsurgical techniques. Future models should integrate a broader range of variables, including patient comorbidities, psychosocial factors, and long-term functional goals. There is a need for a large-scale, prospective, multicentre study, potentially supported by trauma registries, to validate such tools and guide consistent clinical pathways. Additionally, incorporating patient-reported outcome measures (PROMs) into routine practice may help shift the focus from limb viability to long-term quality of life, providing a more holistic measure of success.

Surgeons need to be encouraged to share decision-making with patients and their families, ensuring they are involved from the outset, especially in the context of reduced consciousness and critical illness [30]. Regular updates to national guidelines, such as BOAST and NICE, should reflect emerging evidence and promote multidisciplinary team training and simulation to enhance decision-making in acute settings. As artificial intelligence continues to advance, machine-learning prediction tools may help support decision-making and contribute to cost-effectiveness analyses. However, it is essential to recognise that these technologies serve only as adjuncts and the ultimate decisions must remain with the multidisciplinary team responsible for the patient’s care.

In paediatric complex limb injury, decision-making introduces further nuances. Considerations such as future growth, long-term limb development, and psychosocial impacts may influence the balance between salvage and amputation differently than in adults. Decision-making, therefore, requires careful, family-centred discussions and long-term planning, ideally supported by multidisciplinary teams that include specialist paediatric trauma and rehabilitation expertise. Finally, to support patient-centred care across all age groups, further research is required into the long-term psychosocial and functional outcomes of both limb salvage and amputation, to better inform clinicians and support patients in this life-changing decision.

## Conclusions

CLI in trauma patients can have life-altering consequences, requiring a multidisciplinary approach to ensure the best possible outcome. Given that CLI affects multiple components of the limb, a thorough and systematic assessment is essential. Despite significant advancements in medical science, the decision to pursue limb salvage or amputation remains highly dependent on clinical judgment. While scoring systems and guidelines provide valuable support, they should never be the sole determinant in decision-making.

Ultimately, patient-centred care must remain at the forefront, ensuring that treatment decisions are made collaboratively across specialities while prioritising both the functional and holistic well-being of the patient. Through a balanced, individualised approach, clinicians can strive to achieve the best possible long-term quality of life for those affected by complex limb injuries.
